# Fostering Digital Resilience Among Youth Who Experienced Cyberbullying: Design Thinking Approach

**DOI:** 10.2196/93891

**Published:** 2026-07-09

**Authors:** Nattharat Samoh, Thomas E Guadamuz, Jintavee Khlaisang, Songphan Choemprayong

**Affiliations:** 1 Technopreneurship and Innovation Management Program, College of Interdisciplinary and Integrative Studies, Chulalongkorn University, Bangkok, Thailand; 2 Mahidol Center for Health, Behavior and Society, Faculty of Tropical Medicine, Mahidol University, Ratchathewi, Bangkok, Thailand; 3 Center of Excellence in Educational Invention and Innovation, Department of Educational Technology and Communications, Faculty of Education, Chulalongkorn University, Bangkok, Thailand; 4 Department of Library Science, Faculty of Arts, Chulalongkorn University, Boromrajakumari Building, 8th Fl, 254 Phayathai Road, Pathumwan, Bangkok, 10330, Thailand, +66 2218 4817; 5 Office of Academic Resources, Chulalongkorn University, Bangkok, Thailand

**Keywords:** digital resilience, cyberbullying, design thinking, youth, Thailand

## Abstract

**Background:**

Cyberbullying affects approximately one-third of Thai secondary school students and has been linked to adverse emotional outcomes and mental health vulnerabilities. Strengthening digital resilience, along with critical thinking, empathy, and help-seeking skills, is essential to empower youth to cope with online risks and promote safer digital behavior. Despite growing recognition of cyberbullying as a critical youth mental health issue, few studies have explored how digital resilience interventions can be co-designed with youth and relevant stakeholders to address the contextual and emotional challenges experienced by affected adolescents.

**Objective:**

This study aimed to develop a youth-centered, theory-informed intervention using a design thinking framework to support digital resilience in addressing cyberbullying. The research repositioned youth with lived cyberbullying experiences from passive persons who experienced abuse to active co-creators of the intervention. Grounded in empowerment principles, nudge theory, and the concept of digital resilience, this approach reimagined cyberbullying prevention through empathy-driven innovation and participatory engagement, generating a contextually grounded model for designing cyberbullying interventions in a Global South context.

**Methods:**

We applied a 5-phase design thinking framework: empathize, define, ideate, prototype, and test. Twelve participants were purposively selected as extreme and lead users and key stakeholders. This group engaged in participatory design workshops and included a psychologist, experts, teacher counselors, and LGBTQI+ (lesbian, gay, bisexual, transgender, queer, and intersex) students aged 15 to 17 years with lived experience of cyberbullying. Youth participants were recruited in collaboration with school counselors to ensure diversity of experiences and perspectives. Qualitative data from the workshops were analyzed using reflexive thematic analysis to identify user needs, guide problem framing, and inform prototype development. A low-fidelity mobile-based prototype in the form of screen mockups was iteratively refined through user testing and expert consultation to ensure theoretical alignment and contextual relevance.

**Results:**

The co-creation process identified key challenges, including emotional distress, lack of support, and the rapid spread of harmful content. Youth participants collaboratively designed a virtual community platform featuring peer support spaces, confidential expert consultation, skill-building workshops, a knowledge hub, gamified engagement tools, and artificial intelligence–assisted support. The intervention design was informed by the study’s theoretical framework, which guided the development of engagement strategies, supportive communication features, and youth-centered interaction pathways. Iterative refinement contributed to improvements in perceived usability, personalization, and theoretical coherence. Importantly, the process supported youth agency by positioning participants as active co-designers who shaped solutions grounded in their lived experiences.

**Conclusions:**

This study illustrates how a theory-driven, participatory design thinking approach can be used to develop digital prevention and intervention for cyberbullying. By integrating behavioral theory with youth co-creation, the findings provide insights into how youth-centered co-design can inform the development of interventions addressing cyberbullying, highlighting the role of empowerment-oriented and theoretically grounded design processes in engaging youth as active contributors to solution development.

## Introduction

### Background

#### Cyberbullying Among Youth

The digital landscape offers both opportunities for social interaction and increased vulnerability to harmful behaviors, such as cyberbullying [1,2]. Globally, the United Nations Children’s Fund reported that approximately 33% of youth across 30 countries have experienced cyberbullying [3]. In the Asian region, widespread smartphone use and intensive social media engagement among adolescents have heightened concerns regarding online peer victimization. Emerging evidence indicates that cyberbullying remains highly prevalent among youth in this region [4,5]. In Thailand, reported prevalence ranges from approximately 20% to 49% [6,7], particularly within school environments and among secondary school students. One study found that 1 in 3 Thai secondary school students reported experiencing cyberbullying [8]. While another investigation among high school and university students indicated that 25% to 50% had encountered online harassment [9]. The research indicates that cyberbullying is a significant issue affecting a substantial portion of young people in Thailand. This phenomenon has profound implications for mental health, contributing to anxiety, depression, and, in extreme cases, suicidal ideation among victims [10-13]. One of the critical aspects of addressing cyberbullying is the need to support the mental health and well-being of the victims. It is essential to build the digital resilience of young people, empowering them with the skills, knowledge, and resources to effectively navigate the digital landscape and cope with the challenges they may face [14,15].

#### Significance of Digital Resilience in Empowering Youth to Deal With Cyberbullying

Digital resilience is the ability to adapt and respond effectively to challenges encountered in online environments [14,16], encompassing both technical skills and psychological attributes that empower individuals to manage digital adversities, such as cyberbullying. By understanding, learning, and recovering, individuals can strengthen their digital resilience and better navigate the digital landscape [14,17,18]. This concept has become increasingly vital as educational institutions recognize the need to equip students with tools to navigate the complexities of the digital world safely [19]. Programs aimed at fostering digital resilience emphasize not only awareness and prevention of cyberbullying but also the development of coping strategies and supportive networks that encourage responsible online behavior [14,15]. Understanding cyberbullying and enhancing digital resilience are crucial for fostering a safer online environment, particularly for youth who navigate these challenges daily [18]. As both the digital landscape and the nature of interpersonal interactions evolve, ongoing research and collaborative efforts will be essential in addressing the complexities of cyberbullying [18,20]. The existing research on cyberbullying in Southeast Asia and Thailand highlights the need for further study and intervention to promote digital resilience in the region [21,22].

Building on this perspective, this study integrates digital resilience, empowerment, and nudge theory as complementary theoretical frameworks to address the complex and multidimensional nature of cyberbullying. Digital resilience serves as the overarching goal, focusing on youths’ capacity to cope with and adapt to online adversity [14]. Empowerment functions as an internal mechanism by strengthening agency, participation, and self-efficacy [23,24], while nudge theory operates as a design mechanism that shapes supportive environments and encourages positive behavioral responses [25]. By combining these perspectives, the intervention aims to address both individual and contextual dimensions of cyberbullying and support a more comprehensive and theoretically grounded approach.

#### Design Social Innovation and Empowering Youth Through Participatory Design Approach

Addressing cyberbullying among youth requires a multifaceted strategy that integrates social innovation, technological solutions, and meaningful audience participation [26,27]. In many low- and middle-income countries and Global South contexts, rapid digital expansion has often outpaced regulatory frameworks, digital literacy infrastructure, and accessible mental health services. As a result, young people frequently navigate online risks within environments characterized by limited institutional support and structural inequalities [28]. Within such settings, adopting a collaborative and participatory design approach becomes particularly critical [29-31]. By engaging youth as co-creators rather than passive recipients, participatory design empowers them to become active agents in addressing cyberbullying [32], fostering ownership and contextual relevance in the solutions developed. Co-design ensures that interventions are culturally responsive, locally grounded, and sensitive to the lived realities of adolescents in resource-constrained settings [33,34]. Moreover, this approach strengthens digital resilience by equipping youth with critical awareness, collective efficacy, and adaptive coping capacities. In Global South contexts, where top-down interventions may not fully capture local complexities, participatory social innovation offers a more equitable and contextually embedded pathway toward safer and more inclusive digital environments.

#### Using Design Thinking Approach to Develop Interventions for Youth

Design thinking has emerged as a practical and human-centered approach for addressing complex social and health challenges. Rather than relying solely on expert-driven solutions, it emphasizes understanding users’ lived experiences and co-creating solutions through iterative development [35,36]. Design thinking is defined as a method for identifying human needs and generating innovative solutions through design-oriented tools and ways of thinking [37]. It functions both as a mindset and a set of practices, offering a solution-oriented framework that prioritizes empathy, collaboration, and real-world relevance in developing effective and actionable strategies.

To foster digital resilience and address the issue of cyberbullying among youth, a design thinking approach can be a powerful tool. Design thinking is a human-centered problem-solving methodology that emphasizes empathy, ideation, and prototyping to create innovative solutions [35,38,39]. By involving young people in the design process [31,40], researchers and practitioners can gain deeper insights into their lived experiences, challenges, and needs, thereby generating contextually tailored interventions [33].

Although widely institutionalized in Europe and North America, the integration of design thinking within Southeast Asian educational and policy contexts remains comparatively limited. Youth interventions in the region are often structured through adult-led, top-down approaches in which adolescents are positioned primarily as recipients rather than contributors to developing solutions [41]. Embedding youth directly within the design thinking process challenges this paradigm by recognizing experiential knowledge as a legitimate form of expertise. This participatory positioning enhances contextual relevance, fosters ownership, and supports culturally grounded intervention design [42].

Furthermore, design thinking facilitates collaboration among diverse stakeholders [43], including youth, educators, counselors, and experts. Such multistakeholder engagement strengthens the comprehensiveness of intervention development and ensures alignment with the complex realities of online environments. Integrating design thinking into youth-focused initiatives therefore contributes not only to empathy-driven and collaborative problem solving but also to the cultivation of digital resilience through context-sensitive strategies capable of generating sustainable impact.

### Objectives

This study aimed to describe the implementation of a design thinking process in developing a youth-centered, theory-informed intervention to support digital resilience through a multiphase participatory approach. Framed within the Thai context, the study integrates user-centered design with behavioral insights, nudge theory, empowerment principles, and digital resilience to inform practical and sustainable intervention development. The study contributes to the field of social innovation by offering a theory-driven, empirically grounded, and contextually grounded approach for addressing cyberbullying within educational and social contexts.

## Methods

This study used a participatory design thinking approach to guide the development of a theory-informed intervention addressing cyberbullying and digital resilience among youth. The following sections describe the study design, data collection procedures, participant recruitment, analytic approach, and strategies used to support research rigor and ethical conduct.

### Study Design and Design Thinking Process

#### Overview

Design thinking was adopted as a user-centered approach and served as the guiding framework for developing an intervention to address cyberbullying among youth. It encompassed 5 distinct stages: empathize, define, ideate, prototype, and test [36], each aimed at uncovering user needs and generating contextually relevant solutions. Outputs from each phase were iteratively integrated to inform subsequent stages, ensuring continuity from user insights to intervention development. Each phase was implemented through structured participatory design workshops, with clearly defined activities, materials, and participant roles aligned with the objectives of each stage. While detailed descriptions are provided in the phase-specific sections below, this structure ensured that the workshops functioned as guided, activity-based design processes rather than unstructured discussions.

#### Phase 1: Empathize

The empathize stage aimed to develop a comprehensive understanding of youth affected by cyberbullying. Techniques including interviews and empathy mapping [44] were used to visualize the thoughts, feelings, and actions of participants in a structured way. Interview activities were conducted using structured prompts to guide participants in reflecting on their experiences with cyberbullying, including the nature of incidents, emotional responses, coping strategies, and perceived support needs. Participants first recorded their responses individually using individual note-taking (eg, Post-it notes) and subsequently shared and discussed these experiences within the group. Through active engagement with students, teachers, and subject matter experts during participatory design workshops, these techniques facilitated exploration of youth experiences with cyberbullying, including the context, thoughts, feelings, coping strategies, challenges, unmet needs, pain points, and gain points, thereby laying the groundwork for problem identification. These activities were conducted within facilitated participatory workshops designed to encourage reflection, collaborative discussion, and shared exploration of cyberbullying experiences.

#### Phase 2: Define

In the define stage, insights from the empathize phase were combined to develop a clear problem statement focused on users’ needs. The statement was framed from the perspective of those affected by cyberbullying to ensure that the identified challenges reflected their lived concerns. The research team examined the question, “What are the key and urgent issues that need to be addressed when dealing with cyberbullying?” To prioritize these issues, a 2×2 matrix [36] was applied to assess issues based on their importance and urgency. Participants engaged in a structured prioritization activity in which pain points identified from the empathy maps were first individually generated and then collectively discussed and positioned within the matrix based on perceived importance and feasibility. Guided group discussions were facilitated to support reflection, comparison of perspectives, and consensus-building among participants regarding the most critical and actionable issues. The top-priority issues were then selected based on their positioning within the matrix and used to inform subsequent persona development and ideation processes. Personas [45] were subsequently developed based on insights generated from the empathize phase and the prioritization of key issues within the matrix. Participants worked collaboratively using persona templates to synthesize key user characteristics (eg, roles, behaviors, motivations, and needs) into representative user profiles. These personas were then used to guide ideation and scenario development in subsequent phases, ensuring that design decisions aligned closely with the real experiences and motivations of the target population. Each persona integrated detailed attributes, including demographic characteristics, cyberbullying experiences, online behaviors, and support needs.

#### Phase 3: Ideate

The ideation stage focused on generating diverse ideas and potential solutions through structured creative exploration. This phase was explicitly guided by theoretical frameworks, including empowerment, nudge theory, and digital resilience, which were used as lenses to generate and evaluate design ideas, with additional input from expert participants. “How might we” prompts, derived from the personas developed in the previous phase, were used as guided ideation activities to stimulate multiple perspectives and expand the solution space [38]. Each persona was used as a reference point to frame “How might we” questions, ensuring that idea generation remained grounded in user needs and contexts. After initial idea generation, participants elaborated on selected concepts using structured worksheets or templates, outlining detailed features such as platform selection, user interface design, and overall user experience. These concepts were further developed through scenario-based storytelling activities, in which each group named their concept and described how the intervention would function in practice through step-by-step user interactions, sometimes supported by simple visual materials to enhance clarity. A structured decision matrix was then used as a guided evaluation activity to assess shortlisted concepts based on predefined criteria, including theoretical alignment, clarity and ease of understanding, congruence with youth lifestyles, feasibility for rapid development, and differentiation from existing solutions.

#### Phase 4: Prototype

In the prototype stage [36], key ideas from the ideate phase were selected and translated into design directions informed by insights and personas developed in earlier phases. These design directions were used to develop low-fidelity mobile screen mockups representing initial design solutions. These mockups, initially developed by the research team, were presented to all participants and refined through iterative, collaborative feedback activities, with opportunities for youth participants to provide input on features, perceived usability, and user experience. Participants were then divided into 4 mixed groups (students, teachers, and experts) to ensure manageable group sizes, support active participation, and incorporate diverse perspectives during prototype development activities. Each group engaged in prototype development and refinement activities, including reviewing, modifying, and expanding the mockups. The groups subsequently presented their revised prototype to the others, enabling cross-group feedback and collective discussion. This collaborative process facilitated iterative refinement and assessment of each concept’s relevance and feasibility in addressing the identified problems. The process was also informed by theoretical considerations, ensuring that design features supported youth agency through empowerment, encouraged positive behavioral responses through nudge-based design, and strengthened adaptive coping capacities as part of digital resilience.

#### Phase 5: Test

In the final stage, prototypes were evaluated through qualitative user testing activities with participants, using 2 key techniques: “I like…, I wish…” [38] and “think-aloud” prompts [46]. The “I like…” questions allowed users to highlight features they found beneficial or enjoyable, while the “I wish…” questions encouraged them to suggest improvements or desired features. Researchers facilitated the “think-aloud” method by prompting participants to verbalize their thought processes and rationale during interaction with the prototype, capturing insights into user perceptions and decision-making. These techniques helped identify areas for improvement, ensuring that the design process remained aligned with user needs and providing a strong foundation for the subsequent mobile app development phase. These testing activities were also informed by theoretical frameworks, including digital resilience, empowerment, and nudge theory, to assess the alignment of the prototype with the study’s theoretical framework. As the prototype consisted of low-fidelity screen mockups, feedback reflected participants’ perceptions and anticipated experiences during simulated interaction rather than interaction with a fully developed digital platform. Nevertheless, these testing activities provided valuable insights for iterative refinement and informed the subsequent development of the intervention.

### Data Collection and Setting

The design thinking process in this study involved 12 participants, including 6 students aged 15 to 17 years who identify as gay, bisexual, or transgender and had experienced cyberbullying. The focus on LGBTQI+ (lesbian, gay, bisexual, transgender, queer, and intersex) youth was intentional, as this group is particularly vulnerable to identity-based cyberbullying, enabling deeper exploration of context-specific experiences and support needs. Participants were purposively selected based on design thinking principles, which prioritize engagement with extreme and lead users rather than large sample sizes. In this study, youth participants were selected because of their lived experiences of cyberbullying, representing extreme cases that provide deep insights into the problem space.

In addition, key stakeholders, including 2 teacher counselors with experience in managing cyberbullying cases, 1 psychologist specializing in cyberbullying and LGBTQI+ youth, and 3 experts in empowerment principles, nudge theory, and digital resilience were included to ensure that the design process incorporated contextual, behavioral, and theoretical perspectives relevant to intervention development. This approach aligns with co-design and design thinking methodologies, which emphasize depth of insight, diversity of perspectives, and iterative exploration of solutions through engagement with relevant users.

Data collection was conducted through a series of participatory design workshops incorporating co-design activities. Youth participants were engaged as active co-creators throughout the participatory design workshops, contributing lived experiences, problem framing, idea generation, and iterative feedback across the empathize, define, ideate, prototype, and test phases, with collaborative design activities aligned with each phase of the design thinking framework. This format enabled participants to share experiences while actively engaging in co-creation, solution development, and qualitative user testing, as described in the *Study Design and Design Thinking Process* section.

Three workshop sessions were held, each lasting approximately 3 hours. Each session followed a structured format aligned with the design thinking phases, incorporating guided participatory activities and supporting materials tailored to each stage. The first session focused on the empathize and define phases, the second on the ideate phase, and the third on the prototype and test phases. The initial prototype was developed by the research team and subsequently refined through iterative input from participants. Youth participants contributed to shaping features, suggesting improvements, and evaluating perceived usability. However, they were not directly involved in the final technical development of the intervention. The first 2 sessions took place in a school counseling room to provide a familiar and psychologically safe environment for student participants. The third session was conducted off-campus at the researcher’s institution, offering a change of environment intended to stimulate creativity and support collaborative learning, design, and innovation activities. The study was conducted within a specific institutional context. This context was intentionally selected to explore cyberbullying experiences within a socially and culturally specific setting. Issues of identity, stigma, and peer interaction may shape online experiences differently from other populations.

### Participant Recruitment

The study was conducted in an all-boys secondary school in Bangkok, Thailand. The school was purposively selected based on the research team’s prior engagement with cyberbullying-related research in context and its supportive environment for student participation, creative activities, and student-teacher collaboration. Previous research suggested that the school provided a contextually relevant setting for exploring cyberbullying experiences among LGBTQI+ youth.

Participant recruitment was conducted purposively in collaboration with school counselors who were familiar with students’ backgrounds and support needs. Following school selection, the research team coordinated with school counselors to identify potential student participants through existing student support and counseling activities within the school. Initial participants were selected from students actively engaged in school activities, who then assisted in inviting peers who met the inclusion criteria. Youth participants were selected based on the following criteria: (1) self-identification as LGBTQI+ (including gay, bisexual, and transgender identities), (2) prior experience related to cyberbullying, and (3) willingness to participate in collaborative design activities. To ensure diversity of perspectives, the recruitment process also considered variation in student roles and levels of school engagement, including student leaders, volunteers, and general students. This approach was used to reduce reliance on a single participant subgroup and to capture a broader range of youth experiences and perspectives.

Teacher counselors were purposively recruited based on their professional experience, familiarity with students’ lived contexts, and representation of both male and female perspectives. In addition, 1 psychologist with expertise in LGBTQI+ adolescent mental health and cyberbullying was recruited to provide relevant mental health perspectives for the intervention design process. Three experts representing diverse theoretical and professional backgrounds related to empowerment theory, nudge theory, and digital resilience were also included to support the integration of behavioral, contextual, and theoretical perspectives throughout the participatory design workshops. The psychologist and expert participants were recruited through direct coordination between the research team and their affiliated organizations based on their relevant expertise and experience working with adolescents.

### Data Analysis

Qualitative data generated throughout the participatory design workshops and prototype activities were analyzed using reflexive thematic analysis informed by Braun and Clarke’s approach [47-49]. The analysis aimed to develop an interpretive understanding of participants’ lived experiences, support needs, perceptions, and collaborative contributions to the design of a theory-informed intervention addressing cyberbullying and digital resilience.

Data analysis occurred iteratively across all 5 phases of the design thinking process. During the empathize phase, discussions, empathy maps, and participant reflections were collaboratively reviewed and interpreted to identify patterns related to youth experiences, emotional responses, coping strategies, pain points, and support needs associated with cyberbullying. During the define phase, data generated from prioritization activities, matrix discussions, and persona development exercises were synthesized to support problem framing and the identification of user-centered design directions. During the ideate phase, ideas, design worksheets, scenario narratives, and group discussions were collaboratively reviewed to identify recurring design preferences, user-centered priorities, and theoretically aligned intervention features. During the prototype phase, prototype feedback, group discussions, revised mockups, and participant suggestions were documented and collaboratively examined to identify usability concerns, design refinement needs, and opportunities for improvement. During the test phase, participant responses from “I like…, I wish…” activities, think-aloud verbalizations, and prototype feedback were iteratively interpreted to identify patterns related to perceived usability, user experience, perceived relevance, and suggested improvements. Across all phases, findings and participant feedback informed ongoing iterative refinement of the intervention design.

Data sources included audio-recorded workshop discussions, think-aloud activities, participant reflections, empathy maps, facilitator field notes, prototype feedback, and participant-generated design materials. Audio recordings were transcribed verbatim in Thai prior to analysis. NVivo (version 14; QSR International *Pty Ltd*) software was used to support data organization, coding, and retrieval throughout the analytic process.

Members of the research team repeatedly reviewed transcripts, workshop materials, and participant-generated outputs to support familiarization with the data. Initial codes were generated inductively to capture patterns of meaning related to cyberbullying experiences, emotional responses, coping strategies, support expectations, online behaviors, and proposed intervention features. Coding structures and analytic interpretations were iteratively discussed and refined among the research team throughout the analysis process to support reflexive engagement with the data and strengthen analytic rigor.

Reflexivity was considered an ongoing component of the analytic process. Members of the research team had extensive experience in cyberbullying research and youth-focused participatory work, including long-term engagement with LGBTQI+ youth. In addition, the facilitator leading the design thinking process had substantial experience in participatory facilitation and design thinking activities. Regular reflective discussions were conducted throughout the study to critically consider how prior experiences, assumptions, and researcher interactions may have shaped interpretation and decision-making during analysis and co-design activities [48].

Codes were subsequently organized into broader thematic patterns to identify key concerns, user needs, and design considerations relevant to the intervention development process. Rather than treating themes as pre-existing categories emerging directly from the data, themes were actively and reflexively developed through ongoing interpretation, comparison across participants, and collaborative analytic discussions among the research team [49]. The analysis attended to both semantic content and broader contextual meanings associated with youth experiences of cyberbullying and digital resilience.

Within the participatory design approach, participant reflection and feedback were integrated throughout the workshop process. Findings and design insights generated from earlier phases were revisited and discussed collaboratively with participants during subsequent activities, allowing interpretations and design directions to be refined iteratively in response to participant perspectives and experiences. This process supported the alignment of the intervention design with participants’ lived experiences and contextual needs.

### Addressing Quality of the Research

To support trustworthiness, the study applied the criteria proposed by Lincoln and Guba [50,51], including credibility, dependability, transferability, and confirmability. Credibility was strengthened through engagement with diverse participant groups, including youth participants, teacher counselors, mental health professionals, and theoretical experts, as well as through the use of multiple data sources and participatory activities, such as workshop discussions, observations, prototype feedback, and co-design activities, to support triangulation. Interpretations and design insights generated during earlier phases were also revisited collaboratively with participants throughout subsequent workshop activities to support ongoing reflection and refinement of interpretations [52].

Dependability was supported through the systematic application of the 5-phase design thinking process and detailed documentation of workshop procedures, analytic decisions, and iterative refinements throughout the study. Confirmability was strengthened through collaborative coding and analytic discussions among the research team, the inclusion of participant quotations, participant feedback during workshop activities, and the use of reflexive notes to critically consider researcher assumptions and interpretations during analysis. Transferability was supported through detailed descriptions of the study context [53], participant characteristics, and participatory design processes, enabling readers to consider the relevance of the findings to other contexts involving youth and cyberbullying prevention.

### Ethical Considerations

#### Ethics Approval

The study protocol was reviewed and approved by Chulalongkorn University, Office of the Research Ethics Review Committee for Research Involving Human Subjects (certificates of approval 003/67 and 134/68).

#### Informed Consent

A waiver of guardian permission and written assent was granted for student participants aged 15 to 17 years by the Ethics Review Committee due to the sensitive nature of the study. As participants self-identified as LGBTQI+ youth, requiring signed assent could pose potential risks (eg, unintended disclosure to family or school). Therefore, verbal informed assent was obtained from all students prior to participation. For teachers, psychologists, and expert participants, written informed consent was obtained. All participants were informed about the study purpose, procedures, potential risks and benefits, and their right to withdraw at any time, with opportunities provided for questions prior to participation.

#### Privacy and Confidentiality

Audio recordings were conducted only with prior permission. All data were securely stored in password-protected, encrypted devices accessible only to the research team. Identifiable information was anonymized or replaced with pseudonyms to prevent identification. All data will be securely destroyed upon completion of the study.

#### Participant Compensation

Participants received compensation of 500 THB (approximately US $15) per session for up to 3 sessions, along with refreshments. Participation was voluntary, with no costs incurred.

## Results

### Participant Characteristics

A total of 12 participants were involved in the participatory design workshops. The demographic characteristics of the participants are presented in Table 1 to provide contextual background for understanding the lived experiences, stakeholder perspectives, and collaborative design contributions that informed the development of the theory-informed intervention to support digital resilience among youth.

**Table 1.** Characteristics of study participants.

Group	Age (years)	Gender at birth	Self-identified gender or sexual orientation	Education	Role or background
Student 1	15	Male	Transgender	Grade 10	Active student leader
Student 2	15	Male	Bisexual	Grade 10	Student volunteer
Student 3	15	Male	Bisexual	Grade 10	Active student leader
Student 4	16	Male	Gay	Grade 11	General student
Student 5	16	Male	Transgender	Grade 11	General student
Student 6	17	Male	Gay	Grade 11	Student council president
Teacher 1	34	Male	Not reported	Master	School counselor
Teacher 2	37	Female	Heterosexual	Bachelor	School counselor
Expert 1	35	Female	Heterosexual	Master	Empowerment theory
Expert 2	38	Female	Heterosexual	PhD candidate	Digital resilience
Expert 3	42	Female	Heterosexual	PhD	Nudge theory
Psychologist	37	Male	Gay	Master	LGBTQI+^a^ adolescent health

a LGBTQI+: lesbian, gay, bisexual, transgender, queer, and intersex.

This study used the 5-phase design thinking framework to co-create a cyberbullying intervention with youth and stakeholders. The following sections present key findings from each phase: empathize, define, ideate, prototype, and test. They illustrate how participatory methods contributed to the development of a user-centered solution to foster digital resilience among youth affected by cyberbullying.

### Phase 1: Empathize

The analysis of data from the participatory design workshops revealed several key themes related to the experiences, impacts, and needs of students facing cyberbullying. Students reported experiencing cyberbullying across various popular social media platforms, including Facebook, Instagram, and X (formerly known as Twitter). Types of cyberbullying included discrimination and ridicule based on gender identity, and the creation of exclusive online groups for gossip, harassment, and public humiliation through negative comments and body shaming; the emotional and psychological consequences left students feeling disappointed, sad, and depressed, socially withdrawn, and fearful. In some cases, the pain and anger resulting from cyberbullying fueled a desire for retaliation. When confronted with cyberbullying, students sought support from various sources. Confiding in trusted friends, empathetic teachers, or individuals who could offer support and guidance proved invaluable, some heal themselves, and some choose not to care or say directly that they do n’ot like it.

**Figure 1.**
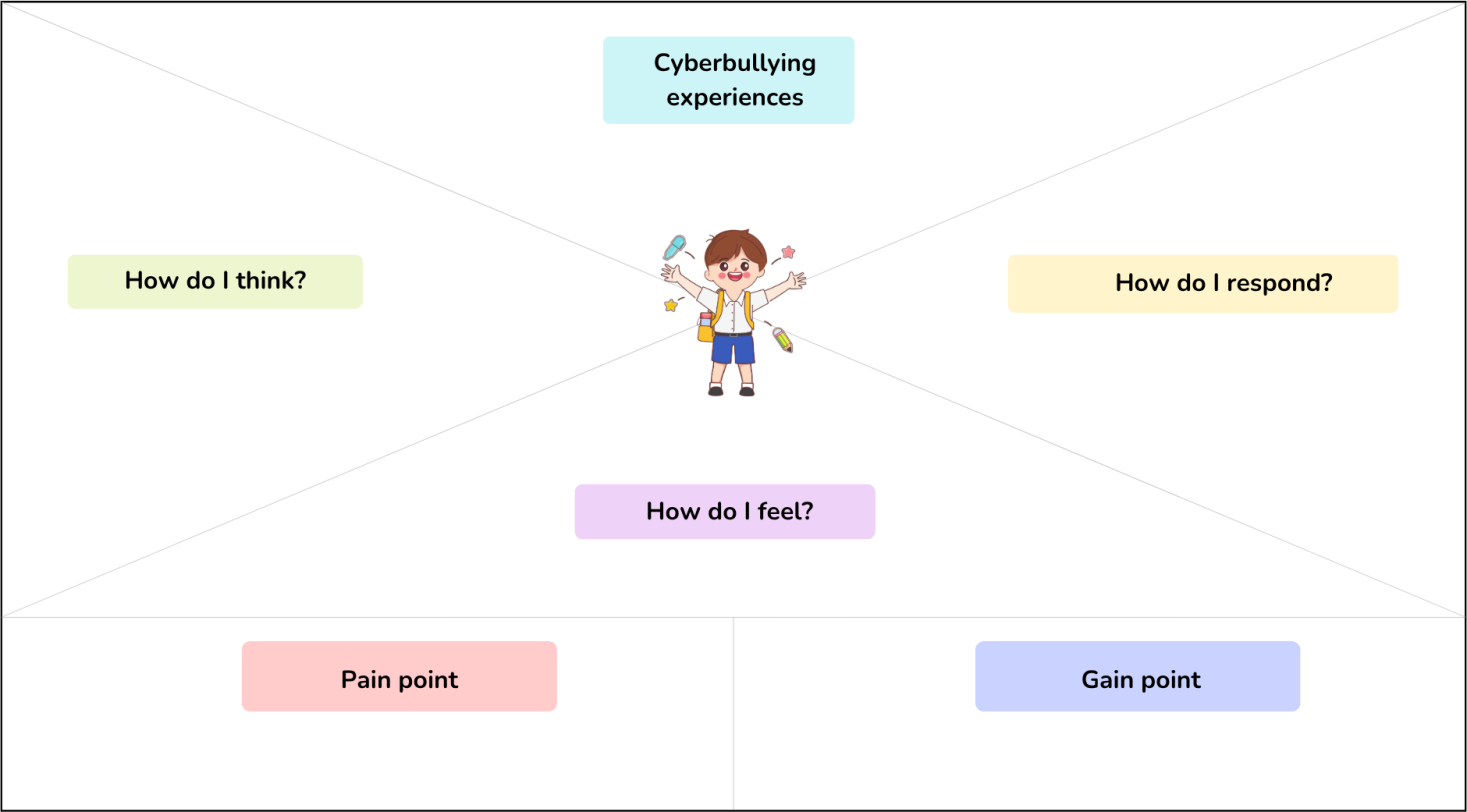
Empathy map template illustrating students’ experiences with cyberbullying.

These findings were further synthesized through empathy mapping and categorized into key dimensions, including cyberbullying experiences, thoughts, feelings, and coping strategies, as well as pain points and gain points, as shown in Figure 1 and Table 2.

**Table 2.** Overview of empathy map findings on students’ experiences with cyberbullying.

Category	Findings
Cyberbullying experiences	(1) Mocked during Instagram Live session; (2) screenshotted and gossiped about in group chats; (3) photo edited to alter gender appearance; (4) shamed for “wanting attention”; (5) excluded from a group chat created specifically for gossip; (6) harassed by fake accounts about hairstyle on Instagram Stories; (7) insulted for appearance and femininity; (8) teased online as “App Man” (mocking an act of masculinity); (9) subjected to sexual harassment.
How do I think?	(1) “At first, it was hard to deal with, but eventually it felt normal”; (2) “I thought the perpetrator was very impolite”; (3) “I wondered what I had done wrong and why I was treated that way”; (4) “Social media increases the risk of miscommunication”; (5) “Those who lack maturity in using social media often seek sadistic pleasure”; (6) “It became something I had to live with”; (7) “Real-life bullying experience makes online bullying easier to handle.”
How do I feel?	(1) Hurt; (2) sad/crying; (3) mood swings; (4) felt depressed; (5) desired revenge; (6) annoyed with that friend.
How do I respond?	(1) Shrugging it off; (2) responding sarcastically; (3) building resilience and self-protection; (4) expressing discomfort directly to the perpetrator; (5) engaging in self-care through social interaction; (6) talking to friends; (7) seeking advice; (8) letting it go (“the world keeps moving anyway”).
Pain point	(1) Being bullied repeatedly; (2) causes misunderstanding; (3) facing perpetrators who ignore our feelings; (4) suffering reduced confidence on social media; (5) keeping the experience to oneself; (6) navigating the uncontrollable spread of harmful content online; (7) choosing not to burden others; (8) feeling that no one understands, and having no one to consult; (9) experiencing bullying from adults or institutional structures.
Gain point	(1) Talking to like-minded friends; (2) engaging in enjoyable activities; (3) having someone to listen; (4) receiving advice; (5) accessing online support groups; (6) consulting teachers available for online or offline counseling; (7) having opportunities to meet others; (8) receiving positive words that aid healing; (9) having someone checking in to ask if they are okay; (10) experiencing a supportive presence; (11) requiring follow-up care for counseling.

### Phase 2: Define

**Figure 2.**
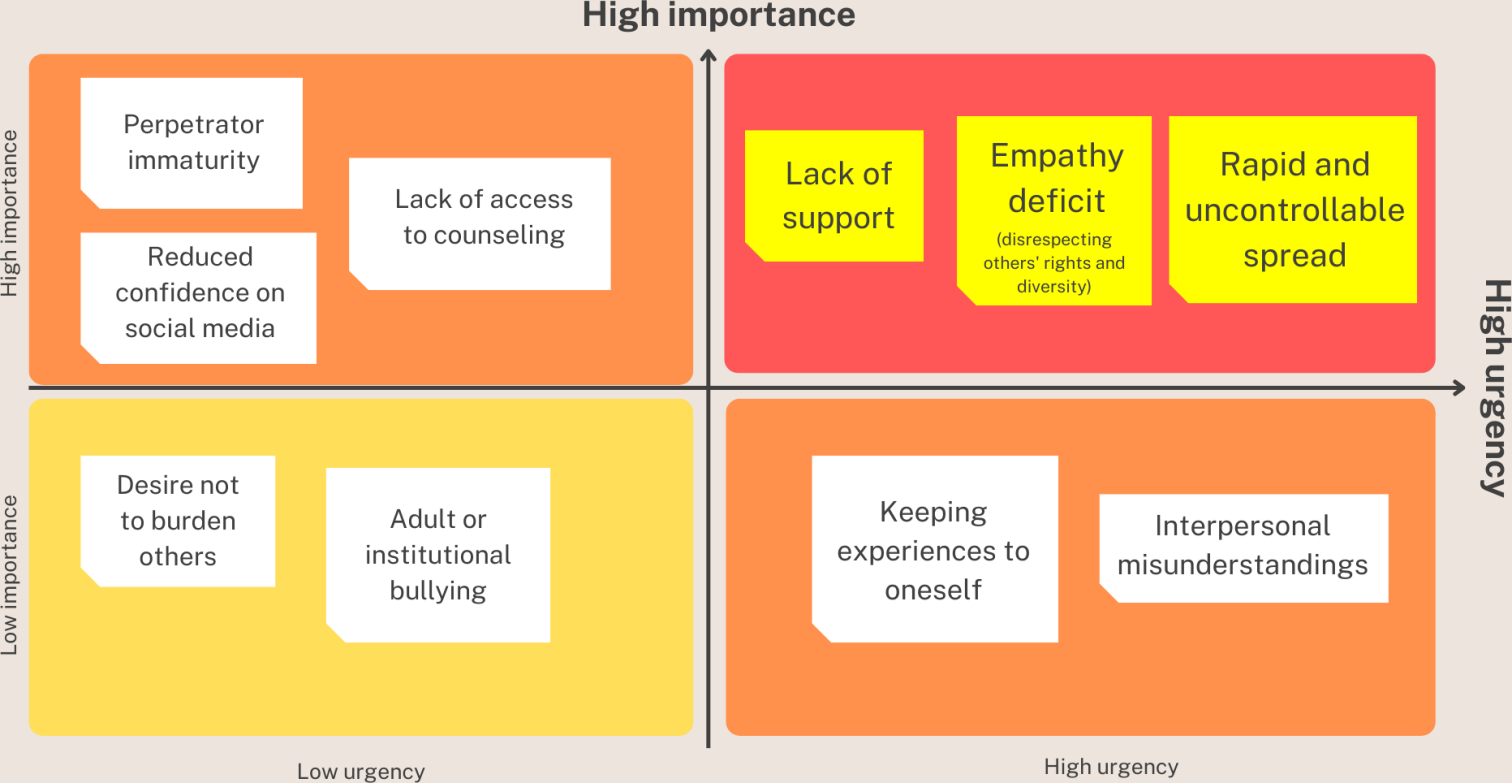
A 2×2 matrix illustrating key aspects of the problem statement related to cyberbullying.

This stage synthesized insights from the empathize stage to identify key challenges in cyberbullying. Participants highlighted several issues, including uncertainty about self-expression, the rapid and uncontrollable spread of the problem, a lack of support or understanding from others, the potential for misunderstandings, the necessity to keep the experience to themselves, and the perpetrators’ failure to recognize the emotional impact on the victims. These findings highlight the complex nature of cyberbullying and the varied emotional and social consequences it has for youth. When these issues were prioritized using a 2×2 matrix based on importance (vertical axis) and urgency (horizontal axis), as shown in Figure 2, the 3 most critical and urgent challenges were identified as (1) victims often lack adequate support and understanding from peers, adults, and institutions; (2) perpetrators frequently fail to recognize or acknowledge the profound emotional impact of their actions; and (3) the rapid and often uncontrollable spread of harmful content online distinguishes cyberbullying from traditional forms of bullying.

Following the identification of the core problem, persona tools were used to design user profiles that captured key aspects such as experiences of cyberbullying, online behavior, motivations, and emotional needs. These profiles enabled the design team to define target users and align intervention features with the authentic contexts and challenges faced by youth. Three distinct personas *Arkla, Keaw, and Maymin* were collaboratively created by research participants. Each persona represents a 13‐ to 15-year-old LGBTQI+ student who experienced cyberbullying. *Arkla,* currently experiencing rapidly spreading cyberbullying, is characterized as a quiet, introverted individual lacking close friends and desiring supportive companionship. Their primary online engagement is through Instagram and X to stay informed about trends and updates from favorite celebrities. *Keaw*, also a victim of cyberbullying, is academically successful and enjoys gaming, yet struggles with the perpetrators’ disregard for their rights and feelings. Keaw desires a supportive network, particularly individuals who share similar gaming interests and can offer both companionship and emotional support through online chats. Finally, Maymin, lacking understanding and support following a cyberbullying incident, desires a trustworthy and empathetic listener. *Maymin*, while cheerful, experiences anxiety and difficulty expressing emotions, finding solace in dance and music, and frequently using platforms such as TikTok, Instagram, and LINE. These personas were designed to capture the diverse experiences of youth, guiding the development of targeted, user-centered interventions. These personas were not intended to directly mirror individual participants but to synthesize shared patterns and user needs into actionable design representations that informed subsequent design decisions. Each persona reflects the experiences of LGBTQI+ youth who have encountered cyberbullying, ensuring that identity-related challenges and context-specific needs remain embedded in the subsequent design process.

### Phase 3: Ideate

Building on insights from the previous phases, the ideation phase generated a wide range of innovative ideas aimed at fostering digital resilience among students to address cyberbullying. Personas developed in the previous phase were used to inform idea generation and scenario development, helping to ensure alignment with user needs and contexts. Using the “How Might We” questioning technique, participants engaged in divergent thinking to explore multiple solution pathways. Importantly, theoretical integration was embedded directly within the ideation process. Experts in empowerment principles, nudge theory, and digital resilience actively participated in the workshops, ensuring that emerging ideas were continuously examined through these theoretical lenses and aligned with the intended behavioral and psychological objectives.

Proposed interventions addressing the lack of support for cyberbullying victims included specialized listener services, artificial intelligence (AI)–based or robot-based emotional support, applications connecting users with empathetic individuals, community platforms for emotional sharing, and online public forums for open dialogue. To mitigate empathy deficits, participants suggested tools for emotional reflection (eg, reflective mirrors and interactive dolls), VR simulations to experience victimization firsthand, moral drama for awareness raising, cyberbullying severity rating systems, peer networks to warn perpetrators, and automated cyberbullying detection notifications. In response to the rapid and uncontrollable spread of cyberbullying, ideas included reporting and content deletion applications, legal measures targeting cyberbullying behaviors, social media content screening mechanisms, cyber surveillance systems, punitive approaches, educational interventions for perpetrators, and structured support groups for victims. Collectively, these diverse proposals reflected a holistic attempt to address the emotional, behavioral, and structural dimensions of cyberbullying. Collectively, these diverse proposals reflected a holistic attempt to address the emotional, behavioral, and structural dimensions of cyberbullying, while remaining theoretically grounded through the active involvement of expert participants, who ensured alignment with empowerment, nudge theory, and digital resilience principles.

Scenario-building and storytelling techniques were subsequently used to elaborate 3 shortlisted concepts in greater depth: The *Cyber Game Simulation* envisioned a situation in which a youth experiencing cyberbullying had no one to consult; within the game environment, users could create avatars, seek guidance from experts, and invite others to collaboratively resolve cyberbullying scenarios.

The *Relationship Puppet Robot* addressed situations in which victims were misunderstood. The robot, designed with human-like interaction capabilities, enabled users to communicate through text, voice, or video. It was programmed to detect keywords and emotional cues and to assist in clarifying misunderstandings by disseminating accurate information to relevant parties while also providing emotional affirmation and encouragement.

The *VEC Community Platform* was designed as a secure virtual space for individuals affected by rapidly spreading cyberbullying incidents. The platform integrated expert guidance, administrative complaint management, peer support, interactive events, and educational sessions within a community-based structure. Each concept was refined with continued input from theoretical experts to ensure conceptual coherence and alignment with empowerment, behavioral guidance, and resilience-building principles.

After defining the key features of the 3 solutions, a structured decision matrix was applied to evaluate the shortlisted concepts based on predefined criteria, including theoretical alignment (digital resilience, empowerment, and nudge theory), clarity, contextual fit, feasibility, and distinctiveness. Voting results from students, teachers, and experts were aggregated, and the *VEC Community Platform* emerged as the winning idea and was selected to advance to the prototyping stage.

### Phase 4: Prototype

**Figure 3.**
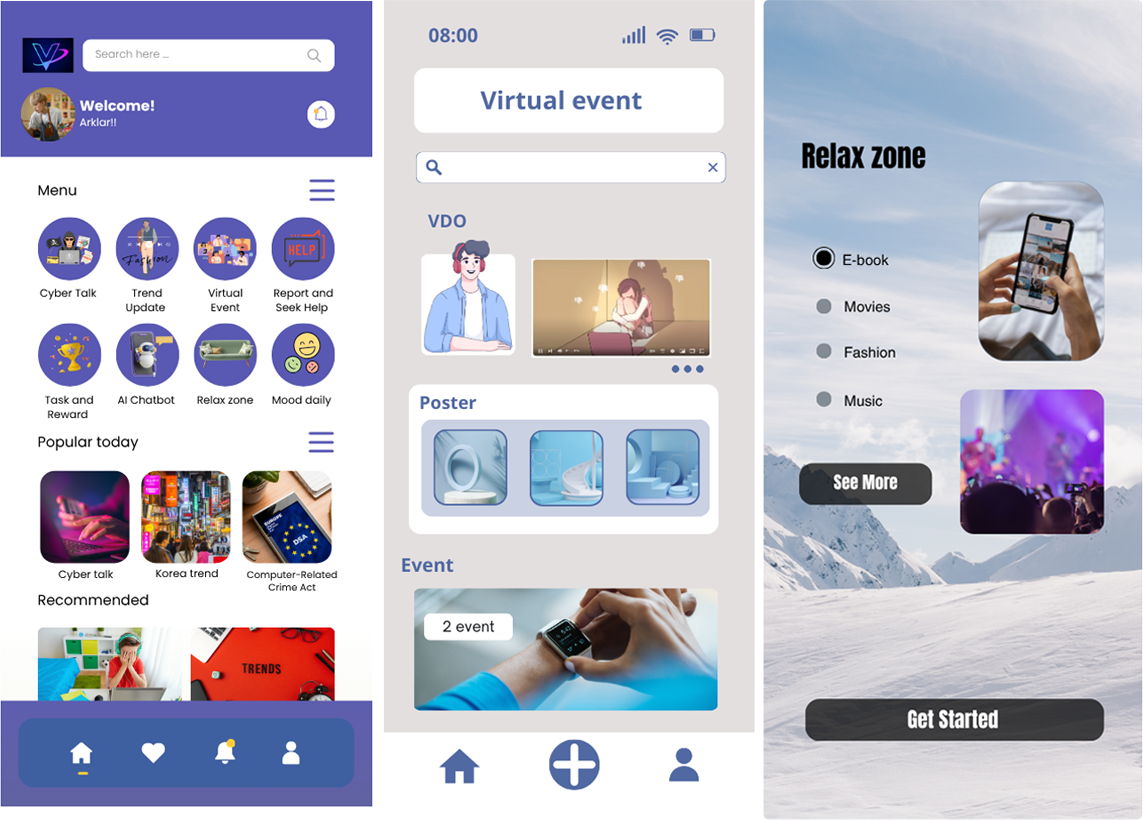
A low-fidelity mobile app prototype in the form of screen mockups for a cyberbullying community platform.

In the prototype phase, a low-fidelity mobile app prototype in the form of screen mockups, as shown in Figure 3, was initially developed by the research team and iteratively refined through collaborative feedback from participants. Youth participants played a key role in evaluating and refining the prototype by providing suggestions on features, perceived usability, and user experience based on their lived experiences. The primary objective of the prototype was to create an interactive and user-friendly platform that addresses the needs of cyberbullying victims while fostering a supportive community. The prototype aimed to facilitate communication among users and provide them with resources to manage and report cyberbullying incidents.

The prototype was developed with an emphasis on simplicity and ease of understanding. Initial sketches and wireframes were created to map out the basic user flow, followed by the development of low-fidelity mockups for key features, such as (1) *Cyber Talk*: a conversation interface allowing victims to communicate with community members, including peers, teachers, counselors, and experts. Users have the option to engage in conversations via chat or video calls, with the flexibility to either reveal or hide their identities; (2) *Trend Update*: a feed displaying a virtual interface to discuss the latest trends, both educational and non-educational topics, such as fashion, music, K-pop, and fiction, as well as news related to cyberbullying, cyber law, digital resilience, and digital safety; (3) *Virtual Event*: a section dedicated to hosting live webinars and online workshops aimed at educating users on various aspects of cyberbullying, as well as organizing fun activities such as video clip competitions; (4) *Report and Seek Help*: a feature that allows users to report incidents of cyberbullying and seek assistance from professionals, including legal support if necessary; (5) *Task and Reward*: a gamified system where users complete tasks related to the platform’s community-building efforts, earning rewards such as badges and coins for their participation; and (6) *AI Chatbot*: an AI-powered chatbot that provides immediate assistance, answers frequently asked questions, and guides users to appropriate resources based on their needs.

In this phase, the initial draft of the cyberbullying community platform was developed using low-fidelity screen mockups to visualize key user flows and interface elements. These mockups allowed participants to understand the overall structure, imagine the user experience, and provide feedback before testing and refinement. Feedback from participants led to the addition of two new features to improve functionality and user engagement: (1) *Relax Zone*, a virtual space offering activities such as fashion exploration, e-book reading, and movie watching, designed to attract a broader audience and enhance platform interaction; and (2) *Mood Daily*, which enables users to reflect on their day by sharing personal stories, providing self-encouragement, and storing these reflections in a “memory jar” for future revisits. These features, derived from participant feedback, aim to promote emotional well-being and foster ongoing platform engagement beyond the core issue of cyberbullying. They also demonstrate the operationalization of theoretical frameworks, with empowerment shaping participatory and peer support components, nudge theory informing behavior-oriented design elements, and digital resilience guiding the overall structure of the intervention. The prototype is presented in Figure 3.

### Phase 5: Test

The testing phase evaluated the low-fidelity mobile app prototype in the form of screen mockups through iterative user testing with 12 participants. The objective was to assess perceived usability, perceived relevance, and perceived appropriateness of proposed features and to identify areas for refinement. Participant feedback, particularly from youth, informed the iterative refinement of the prototype and contributed to its alignment with user needs. As the prototype consisted of screen-based mockups, user feedback reflected participants’ perceptions and experiences during simulated interaction rather than interaction with a fully functional system.

Overall feedback indicated strong engagement with the platform’s structure and menu-based navigation. Participants appreciated the customizable interface and intuitive layout, while suggesting greater flexibility in content personalization. Feature-specific insights revealed the importance of avatar personalization and context-sensitive privacy settings within the *Cyber Talk* function.

The *Trend Update* feature was perceived as valuable for reducing feelings of isolation and promoting peer interaction, particularly when content reflected current and relevant topics. Suggestions for the Virtual Event function emphasized visibility, diverse content formats, and the involvement of influential speakers to enhance participation.

Feedback on the *Report and Seek Help* feature highlighted the need for 24-hour availability, structured complaint prioritization, and streamlined search functionality. The *Task and Reward* system was positively received, with participants favoring intrinsic, community-based recognition over material incentives.

The *AI chatbot* was viewed as accessible and supportive, particularly for immediate and anonymous consultation. However, concerns were raised regarding emotional sensitivity and the need for more empathetic response mechanisms. Illustrative participant quotations supporting these findings are presented in Table 3.

**Table 3.** Illustrative participant feedback during screen-based mockups prototype testing.

Feature and key insight	Participant quotation
Menu customization	
Need for flexible personalization	“Some users might want a ’popular today’ section, while others may not, or they might prefer a ’recommended’ section or choose not to include it.”
Overall interface	
Supermarket-like navigation experience	“I like it. It gives the feeling of walking in a supermarket, where we can choose which store we want to go into, similar to selecting a menu.”
Cyber talk	
Desire for more personalized avatars	“I like this because I already enjoy using Gather Town. Actually, I think the avatars could be even cuter. I want each character to represent the person they are in real life.”
Context-based video settings flexibility	“As for the video settings, I would suggest the default be set to camera on, with the option to turn it off. However, if the conversation is with a psychologist, I would prefer the default to be camera on, without the option to turn it off. But for conversations with friends or classmates, it should be possible to turn off the camera.”
Trend update	
Reduces isolation and promotes discussion	“It would make us feel less isolated or lonely. This group may not just be for counseling; we could also talk about various random topics. It could be a group conversation because everyone has different thoughts, and we could continue to brainstorm ideas together.”
Importance of trending content	“The topic needs to be trending at the moment. When we enter the conversation, if it’s about something that’s currently trending, it will allow the discussion to continue for a longer period.”
Virtual event	
Trend-driven, influencer-led events to enhance user engagement	“It could be an event where people join to follow current trends or updates. Influencers or popular creators might host live sessions, similar to TikTok live streams where someone plays games and many viewers watch. Some may join to relax, relieve stress, or simply look for news and interesting content.”
Report and seek helSeek Help	
Need for 24-hour service availability	“I would like the service to be available 24 hours a day because we never know when someone might encounter a cyberbullying issue. Some people may face problems at night, and if the system is closed, it becomes difficult to find help at that time.”
Task and reward	
Intrinsic, avatar-based rewards to enhance motivation	“If it’s a voucher, some people might feel too lazy to redeem it. They may be more attracted to rewards like avatar decorations. For example, they might want to stand out from others, so their avatar outfit should be different, such as wearing a crown.”
AI chatbot	
24-hour consultation support	“In the case of consulting with a real person, they are not online 24 hours a day. But with this, it is available 24 hours, which helps provide some level of support.”
Safe space for emotional expression	“It feels like we can talk about anything without the person being able to remember it. It’s like venting. If we talk one-on-one with a friend, there’s always the possibility of them gossiping about us behind our back. Having a chatbot is beneficial because some people may not trust other humans.”

Overall, the findings presented in Table 3 indicate key user needs related to flexible personalization, safe and context-sensitive communication, timely support, and engaging community-based interactions. The results further demonstrate that features such as *Trend Update* and *AI chatbot* address users’ coping needs and experiences of isolation, *Cyber Talk* facilitates user agency and interactive engagement, and the *Task and Reward* system supports user motivation and sustained participation, reflecting the integration of digital resilience, empowerment, and nudge theory within the intervention design.

### Intervention Refinement After Testing

Design thinking in this study was not treated as a linear process with a definitive end point, but rather as an iterative cycle of refinement. Insights generated during the testing phase were not considered final outcomes; instead, they informed successive rounds of redesign conducted in collaboration with stakeholders, including academic experts, teachers, students, and a psychologist. The prototype underwent multiple revisions in which theoretical frameworks were operationalized to guide design decisions, ensuring alignment with a theory-driven framework grounded in empowerment principles, nudge theory, and the concept of digital resilience. This continuous integration of user feedback and theoretical guidance strengthened both the conceptual rigor and practical relevance of the intervention.

The outcome derived from the design thinking process, which will be implemented in the subsequent phase of the study with student participants, is a virtual space designed to foster digital resilience and support adolescents affected by cyberbullying. The platform incorporates 5 core components. First, a peer support space enables students to connect through video, voice, or chat, fostering solidarity, empathetic listening, and normalization of help-seeking behavior. Second, a safe space for expert support provides confidential access to psychologists, teachers, and youth counselors, offering professional guidance and emotional support. Third, virtual workshops for skill development promote digital literacy, media awareness, and self-efficacy through participatory learning sessions. Fourth, a knowledge hub and resource center delivers accessible educational materials on online safety and cyberbullying prevention, encouraging informed digital citizenship. Finally, games and interactive quizzes introduce gamified elements that enhance engagement, emotional relaxation, and informal learning within a supportive community environment [54].

Importantly, the relationship between platform features and theoretical foundations is not linear or mutually exclusive. Rather than assigning each component to a single theory, the intervention functions as an interconnected ecosystem in which empowerment, behavioral nudging, and resilience-building dynamically reinforce one another. The peer support space strengthens social connectedness and collective efficacy, reflecting empowerment principles while simultaneously enhancing digital resilience by reducing isolation and fostering adaptive coping. The safe space for expert support increases perceived control and access to guidance, contributing to both psychological empowerment and emotional regulation.

Similarly, the virtual workshops and knowledge hub operationalize digital resilience by cultivating critical thinking, media literacy, and informed decision-making while also reinforcing agency and self-efficacy. The gamified features, informed by nudge theory, embed subtle behavioral cues and positive reinforcement within engaging activities. Beyond influencing behavior, these mechanisms promote reflection, emotional awareness, and prosocial norms, indirectly strengthening both empowerment and resilience capacities.

Taken together, the intervention can be understood as an integrated digital design in which social support, skill development, and behavioral guidance are combined. Through iterative refinement and theoretical integration, the platform reflects a psychologically informed and contextually responsive approach to supporting adolescents experiencing cyberbullying.

## Discussion

### Design Thinking Approach in Developing Cyberbullying Intervention

This study demonstrates how the design thinking approach can be a powerful methodology to develop youth-centered interventions that are both empathy-driven and contextually grounded. Its application represents a paradigm shift toward user-centric innovation, especially effective for addressing complex issues such as cyberbullying among youth [55]. The design thinking’s 5-stage process, empathizing, defining, ideating, prototyping, and testing [38], ensures that interventions are not only innovative but also firmly grounded in the lived realities of the target population [56]. In this study, engaging youth as co-designers transformed them from passive recipients into empowered stakeholders, fostering ownership and increasing the likelihood of adoption and long-term impact [57,58].

By placing empathy at the heart of the process, the intervention team was able to gain a deep understanding of the emotional, psychological, and social challenges faced by cyberbullying victims. These insights informed the development of tailored features within the intervention, such as *Cyber Talk*, *Virtual Event*, and *Trend Update* that resonated with users’ needs and experiences [59]. In the prototyping and testing phases, the iterative feedback loops ensured continual refinement of the intervention. This not only improved usability and engagement but also reinforced a sense of ownership among the participants, which is critical for the long-term sustainability of social innovations [60]. As a result, the final mobile-based intervention was not only user-friendly and engaging but also emotionally attuned and sustainably designed to meet the real needs of youth affected by cyberbullying [55].

Participant feedback highlighted the empowering nature of the design thinking process. Brainstorming activities were particularly appreciated, as they encouraged creative thinking and exploration of unconventional solutions to cyberbullying. The variety of activities fostered structured thinking and introduced new problem-solving approaches. However, participants noted that deeper engagement would require more time, particularly for iterative refinement. They also suggested involving peers from more diverse backgrounds, such as students from other schools, to broaden perspectives and enhance inclusivity. To evaluate the effectiveness of the design thinking process, it is essential to assess not only the outcomes of the intervention but also the quality of user participation and engagement throughout its development.

While design thinking offers an engaging and participatory framework for innovation, it also presents several challenges. The approach often emphasizes creativity over feasibility, resulting in prototypes that lack technical, financial, or contextual adaptation [61]. Moreover, design thinking is time and resource-intensive, posing practical challenges in school and community settings with limited instructional time and material support [62]. Cultural factors may also inhibit open collaboration and idea-sharing, particularly in hierarchical educational environments common in many Asian contexts [63]. These challenges highlight the need for careful adaptation, structured facilitation, and cultural sensitivity when applying design thinking in youth-centered innovation. Therefore, although it is a valuable methodology, its effective implementation should be supported by complementary frameworks, such as systems thinking and inclusive participation, to ensure sustainability and to address complex real-world problems through multidisciplinary collaboration [60]. In the context of this study, it is important to note that this study represents a participatory phase within a broader design process, rather than a full end-to-end co-design implementation.

Although design thinking has been increasingly applied in digital mental health innovation, its use in cyberbullying interventions remains relatively limited. Prior research has used design thinking to address youth mental health concerns and digital well-being [64,65], demonstrating its value in developing user-centered and technology-enabled solutions. However, cyberbullying presents distinct sociodigital complexities involving peer dynamics, platform structures, and online behavioral norms. Applying design thinking in this context therefore requires integrating psychological, social, and technological dimensions simultaneously.

Furthermore, while the d.school’s 5-stage model [38] is widely cited, alternative frameworks offer complementary perspectives. For example, Scholten and Granic [35] introduced 3 core tenets of design thinking to address limitations in digital mental health interventions for youth: empathy, multidisciplinary ideation, and experimentation. Their work highlights how design thinking can help overcome persistent challenges, including low engagement, limited personalization, and implementation fidelity. By emphasizing cross-disciplinary collaboration and continuous testing, this framework positions design thinking not merely as a creative method but as a structured approach to enhancing the appeal, effectiveness, and scalability of digital interventions.

The present study aligns with this broader conceptualization by integrating empathic inquiry, collaborative ideation, and iterative refinement within a theory-informed structure. In doing so, it extends the application of design thinking to the cyberbullying domain and contributes to a more context-sensitive and participatory model of digital mental health innovation for youth.

The findings of this study should be interpreted within the specific cultural, educational, and social context in which the research was conducted. The study involved LGBTQI+ youth in a Thai secondary school setting, where issues of identity, stigma, and peer dynamics may uniquely shape experiences of cyberbullying and help-seeking behaviors.

These contextual factors may influence the applicability of the intervention in other settings, particularly in environments with different cultural norms, institutional structures, or levels of social acceptance toward LGBTQI+ individuals. Therefore, the findings are not intended for statistical generalization; rather, they offer transferable design insights that may inform the adaptation of similar interventions in comparable contexts.

### Importance of Theoretical Background to Design Intervention

While many applications of design thinking in digital health and youth innovation prioritize user-centered creativity and rapid prototyping, they often lack explicit theoretical integration. Interventions developed through design thinking are frequently driven by experiential insights and iterative testing, without being systematically anchored in established behavioral or psychological frameworks. This study addressed this gap by deliberately embedding a theory-driven foundation within the design process, thereby strengthening conceptual rigor, ensuring behavioral coherence, and supporting the potential for sustained impact.

The strength of the intervention lies in its theoretical foundation, drawing from empowerment principles [23,24], nudge theory [25,66], and the concept of digital resilience [14,15,17]. These frameworks shaped the intervention’s structure and strategic focus, addressing cyberbullying as a multilayered phenomenon.

Empowerment theory emphasizes increasing individuals’ sense of control, competence, and social connectedness [23,67]. By involving youth in decision-making and co-creation, the intervention supported psychological empowerment, fostering critical awareness and encouraging proactive behavior in digital contexts. Empowered youth are more likely to stand up for themselves and others online, challenge harmful narratives, and advocate for safer digital spaces [68,69].

Nudge theory, as introduced by Thaler and Sunstein [25], provided a behavioral science lens to subtly guide users toward healthier digital habits. The mobile app used nudging techniques, such as motivational notifications, gentle reminders to pause before posting, and suggested actions for responding to online harassment [66,70]. These features encouraged safer online behavior without being intrusive, preserving user autonomy while fostering reflection and resilience [71,72].

The concept of digital resilience served as the overarching objective, preparing youth not only to withstand the negative effects of cyberbullying but also to adapt, recover, and thrive in the digital world. Digital resilience entails developing emotional regulation, critical thinking, self-efficacy, and help-seeking behavior, skills that were embedded in both the content and delivery of the intervention [14,73,74]. This holistic theoretical integration ensured that the intervention addressed the multidimensional realities of cyberbullying, strengthening youth behavioral agency, emotional resilience, and social empowerment in digital contexts.

### Technology as an Empowerment Tool

Delivering the intervention through a mobile-accessible virtual community platform was a decision that emerged from the ideation and prototyping phases, reflecting participants’ digital habits and communication preferences [75-77]. Participants emphasized flexibility, privacy, and accessibility as key elements for youth who may hesitate to seek face-to-face help. Mobile accessibility also enabled the intervention to integrate into adolescents’ everyday routines, which participants perceived as reducing stigma and facilitating help-seeking [78,79].

The platform incorporates peer support spaces, confidential expert consultation, skill-building workshops, a knowledge hub, gamified engagement features, and AI-assisted support. These elements were designed not only for information delivery but also to support emotional expression and community interaction. During participatory design workshops, youth highlighted the importance of anonymity and identity flexibility; therefore, customizable avatars were incorporated to allow self-expression while minimizing fear of judgment. Mobile accessibility also supported broader reach, particularly in contexts where smartphones function as the primary point of internet access for young people [80]. In many Southeast Asian and Global South settings, a mobile-accessible platform aligns with existing digital infrastructures [81] and extends psychosocial support beyond traditional institutional environments. This expanded access may strengthen equity in cyberbullying prevention efforts [80,82,83].

At the same time, reliance on mobile-mediated interaction requires careful consideration. Excessive screen time may contribute to digital fatigue or reduced offline engagement [84], and safeguarding privacy and data security remains critical when engaging minors in virtual environments [85,86]. These considerations underscore the importance of combining digital delivery with human facilitation [87] and ethical oversight to ensure that technology functions as an empowering infrastructure rather than a substitute for meaningful social connection.

In this context, the use of *AI chatbot* features also raises important ethical considerations, including the risk of inaccurate responses, overreliance on automated support, and potential reductions in help-seeking from human professionals [88]. In this study, the chatbot is conceptualized as a supplementary tool intended to provide general guidance and encourage users to seek appropriate human support when needed. Future development should further consider safeguards related to transparency, accuracy, and responsible AI use.

### Overall Implications

This study illustrates the synergistic potential of integrating design thinking methodology, theory-driven intervention design, and a mobile-accessible virtual community platform to address the multifaceted challenges of cyberbullying among youth. By repositioning adolescents with lived experience from passive recipients to active co-creators, the design thinking process ensured that empathy and experiential knowledge were embedded at the core of intervention development, enhancing contextual relevance and usability. The deliberate integration of empowerment principles, nudge theory, and digital resilience provided a robust conceptual foundation that strengthened the intervention’s structural coherence and behavioral impact. Furthermore, the development of a mobile-accessible virtual community platform enhanced accessibility, scalability, and sustained engagement, particularly important for reaching youth in diverse and resource-constrained settings.

Nevertheless, these strengths must be considered alongside methodological and practical limitations. Design thinking, while participatory and user-centered, may encounter challenges in translating creative ideas into feasible and sustainable solutions and often requires substantial time, facilitation capacity, and institutional support. Similarly, reliance on digitally mediated platforms necessitates careful attention to digital fatigue, privacy and data security concerns, and potential screen overuse. These considerations underscore the importance of contextual adaptation, ethical safeguards, and complementary offline support structures when implementing youth-focused digital interventions. Overall, this study offers a replicable and theoretically grounded framework intended to support digital resilience, strengthen youth agency, and promote safer online engagement among adolescents.

Building on these contributions, this study represents an initial phase within a broader research program focused on developing and evaluating interventions to address cyberbullying among youth. While this study emphasizes the design and prototyping process, the insights and intervention developed in this phase have informed subsequent work. In the next phase, the intervention will be further examined using a quasi-experimental design to assess its effectiveness among the target group. The study’s focus on LGBTQI+ youth enabled in-depth insights into identity, stigma, and support-seeking behaviors in cyberbullying contexts. While grounded in this group’s experiences, the findings highlight broader patterns of user needs and may inform contextually appropriate support strategies for other adolescents. However, these insights are shaped by the specific social and cultural experiences of LGBTQI+ youth and may not fully represent the experiences of all adolescents affected by cyberbullying.

### Limitations

This study has several limitations. The design thinking process in this study was conducted across 3 sessions totaling 9 hours, which limited the depth of participant engagement and reduced opportunities for iterative refinement. In addition, the relatively small sample size and purposive selection of participants may limit the diversity of perspectives captured. However, consistent with design thinking approaches, the study prioritizes depth of engagement with extreme and lead users over representativeness.

Second, the study was conducted within a specific institutional and cultural context, which may limit the applicability of the findings to other populations or settings. Although youth participants contributed actively during the design process, including idea generation and usability feedback, they were not directly involved in the final technical development of the intervention. This may have limited their sense of ownership and reduced opportunities for deeper empowerment through hands-on implementation. In addition, the study primarily focuses on evaluating the process of youth participation in generating actionable design insights, rather than developing a fully functional final product.

Moreover, translating student-generated ideas into functional prototypes was limited by a lack of technical resources and educator support, highlighting the need for more integrated, school-supported implementation models in future applications. Finally, this study did not evaluate the effectiveness of the intervention, as it focused on the design, prototyping, and qualitative user testing process, which provided critical insights for developing a feasible and contextually relevant solution for cyberbullying among youth.

### Future Research

This study aims to provide transferable design insights, particularly for similar contexts where youth experience cyberbullying within constrained social or institutional environments. Future research should explore the adaptation, evaluation, and applicability of the intervention across more diverse cultural, educational, and social contexts.

Future research should explore the adaptation, evaluation, and applicability of the intervention across diverse contexts. Ongoing work includes a quasi-experimental study to assess its effectiveness in improving digital resilience and cyberbullying-related outcomes.

Future studies may also further explore the integration of design thinking as a learning and empowerment process, engaging youth in iterative, hands-on activities from empathy-building to prototyping and testing. Such approaches may strengthen digital resilience while fostering agency, creativity, and social responsibility among young people.

However, design thinking is not the only human-centric innovation framework suitable for young learners. Future studies should also explore other participatory and learner-centered approaches such as the Double Diamond model [89], which emphasizes divergent and convergent thinking, STEAM4INNOVATOR, a curriculum-based process fostering entrepreneurial mindsets [90], and systems thinking, which supports holistic understanding of complex, interrelated problems [91,92]. These frameworks complement design thinking by promoting critical analysis, structured creativity, and systems-level insight.

By integrating diverse, youth-appropriate innovation models, future research can advance more inclusive, sustainable, and contextually grounded interventions. Such approaches are especially vital for addressing multifaceted social challenges such as cyberbullying, where emotional, cultural, and technological dimensions intersect. A hybrid framework may yield greater impact by accommodating different learning styles and institutional contexts while still centering youth voices throughout the innovation process.

Future directions in cyberbullying prevention should align with broader goals of promoting digital citizenship and fostering positive behavioral change among youth. Strategies should integrate emotional support, peer engagement, and gamified learning to cultivate empathy, responsibility, empowerment, and resilience. Collaboration between educators, mental health professionals, and policymakers is essential to create safe and empowering digital spaces for young people. Ultimately, a forward-looking approach that combines digital resilience, participatory design, and social innovation holds great potential for building a safer and more inclusive online ecosystem.

### Conclusions

This study illustrates the potential of integrating a theory-driven design thinking approach within a participatory framework to address cyberbullying among youth in a Global South context. By repositioning adolescents with lived experiences of cyberbullying as co-creators rather than passive recipients, the intervention development process supported youth agency while enhancing contextual and cultural relevance. The integration of empowerment principles, nudge theory, and digital resilience provided a structured theoretical foundation that contributed to the conceptual coherence and practical orientation of the resulting digital intervention.

Beyond developing a prototype, this study offers a model for theory-informed, youth-led digital mental health intervention development. It highlights the importance of embedding behavioral science within participatory design processes to inform the development of context-sensitive interventions addressing the complex sociodigital realities faced by adolescents.

## Abbreviations

AI: artificial intelligence
LGBTQI+: lesbian, gay, bisexual, transgender, queer, and intersex
